# Social Capital and Autism in Young Adulthood: Applying Social Network Methods to Measure the Social Capital of Autistic Young Adults

**DOI:** 10.1089/aut.2019.0058

**Published:** 2020-09-03

**Authors:** Elizabeth McGhee Hassrick, Collette Sosnowy, Laura Graham Holmes, Jessica Walton, Paul T. Shattuck

**Affiliations:** ^1^ Lifecourse Outcomes Program, A.J. Drexel Autism Institute, Drexel University, Philadelphia, Pennsylvania, USA.; ^2^ Department of Medicine, Brown University, Providence, Rhode Island, USA.; ^3^ Clinical Mental Health Counseling, Lock Haven University, Lock Haven, Pennsylvania, USA.

**Keywords:** social networks, duocentric networks, social capital, autism spectrum disorders, transition to adulthood, parent networks

## Abstract

Social isolation is a core challenge associated with autism. Interpersonal relationships and the resources and support embedded in the social networks of autistic young adults could impact key adult outcomes, including quality of life, mental health, employment, and independence. However, little research systematically measures the networks of autistic young adults and network impact on key adult outcomes. This article demonstrates how social network analysis can be adapted for the field of autism to measure young adult networks. We provide examples as to how this approach could be implemented to yield key insights into the amount and quality of interpersonal relationships and the types of resources embedded in the networks of autistic young adults. The network protocol was feasibility tested with autistic adults during the posthigh school transition period (*n* = 17, 19–27 years). The parents of three of the recruited young adults also successfully completed a complementary network survey, allowing for the inclusion of the parent-reported network using duocentric network analysis, never before applied to parent–child networks. The implementation data collected from the study suggest feasibility of egocentric and duocentric approaches, with several important modifications to adapt the measure for the field of autism. The future potential of social network research for understanding autism in adulthood is discussed.

## Lay summary

**What was the purpose of this study?:**

Many autistic young adults are disconnected from people, communities, and organizations that could provide them with valuable social resources to support their transition to adulthood. This study tests the feasibility of using social network methods to measure the resources that autistic young adults gain from their social connections. Future studies using our social network measure might provide valuable information about possible interventions that could help autistic youth acquire the social resources needed for successful adult outcomes.

**What did the researchers do?:**

To test the feasibility of our social network measure, 17 autistic adults, between the ages of 19 and 27 years, were recruited to complete a survey about their social connections and the resources provided to them by their social connections. The parents of three of the autistic adults were also surveyed to provide information about the social connections that they relied on to help their autistic young adult to transition successfully.

**What were the results of the study?:**

All participants successfully completed our survey online. We analyzed the network data to illustrate what types of information the survey produced. We demonstrated how the survey captures different types of support that people gained from their networks. We also used network pictures to show how network measures characterize connections among supporters of autistic young adults. This research successfully lays the groundwork for future studies to test whether the social resources young adults on the autism spectrum receive from their networks impact their adult outcomes.

**What do these findings add to what is already known?:**

We know very little about the social networks for young adults on the autism spectrum. Also, no studies have used duocentric network analysis to measure the social capital of youth and their parents together. This project produced new useful ways of collecting social network data from young autistic adults that will produce knowledge about how to help young adults on the autism spectrum build networks that will yield the social resources needed to support positive adult outcomes.

**What are potential weaknesses of this study?:**

The study was only designed to test the feasibility of the social network measure and does not provide generalizable information about how networks might impact outcomes. Also, the social network measure only allowed participants to identify five people, possibly under-reporting the actual size and composition of autistic young adult networks.

**How will these findings help young adults on the autism spectrum now or in the future?:**

If future research suggests that social network factors help young adults on the autism spectrum to improve outcomes, then network interventions, guided by social network data, can be created.

## Introduction

The social isolation of people on the autism spectrum is a concern shared by multiple stakeholder groups including autistic advocates, caregivers, and providers. Across societal institutions including schools, health care organizations, businesses, and community organizations, stakeholders share a common challenge to create inclusive social contexts wherein autistic people have opportunities to forge high-quality interpersonal relationships. However, many autistic young adults are disconnected from contexts beyond the home where they could create meaningful social ties with peers, community members, or service providers. In the United States, about half of young adults on the spectrum are not engaged in postsecondary schooling or employment in the first 2 years after high school.^1^ Their rate of disconnection from these adult social contexts is higher than for young adults with most other disabilities.^1,2^ This represents a significant loss of opportunities to establish interpersonal relationships with friends, romantic partners, mentors, coaches, service providers, and colleagues, and to experience positive social outcomes such as belonging and social integration. Disconnection also restricts access to valuable social capital that can help young adults achieve key adult outcomes, such as employment.^3–7^

### Egocentric and duocentric network methods

Social network methods can be used to characterize the degree and quality of social connections and access to social capital for autistic young adults and determine possible associations with adult outcomes. The structural, normative, and resource dimensions of social capital have been successfully established across fields of study including sociology, education, organizations, criminology, and public health.^8–11^ For example, decades of research suggests that strong ties between the people closest to young adults can create “network closure” that yields support, trust, encouragement, and goodwill for the transition to adulthood.^5,12–19^ Examples of social capital include affective resources, such as collective support for seeking employment or shared expectations about completing college and finding and keeping employment, as well as instrumental resources, such as coordinated logistical supports and targeted financial resources for job coaching. The weak ties that young adults have with acquaintances can also “broker” social connections to valuable resources, such as new employment opportunities^12,13^ and critical resources for seeking and securing employment.^13–15^

The two main types of network analyses are whole network analysis and egocentric network analysis.^20^ Whole network approaches inventory the connections present within a specified group of people, where each person in a specified group reports his or her relationships with others in the group. Whole network approaches can be used to determine the centrality or isolation of autistic young adults among a particular group of peers or coworkers. In contrast, egocentric network approaches ask a person, called the “ego,” to report on people who are important to them (“alters”). They report on their perceptions of the connections among alters and the resources they access from their networks. In addition, key alters who have a significant impact on a person's network (e.g., a parent or mentor) can also complete an egocentric network of their own about people who help them support the original “ego.” The two egocentric networks can be combined into one joint duocentric network to more accurately capture the ecosystem of connections that are important for an autistic young adult.^21^ To date, no studies have used egocentric social network methods to capture the ecosystem of important people for young adults on the spectrum. Duocentric network approaches, never before applied to parent–/mentor–child pairs,^22,23^ could be used to allow for the inclusion of network reports from parents or mentors about the important people they seek out to support the young adult. This article illustrates how to use duocentric network methods to map ecosystems for young autistic adults.

### Social networks and young adults on the autism spectrum

Building relationships is challenging for many adults. Autistic young adults face additional persistent challenges with social communication (Diagnostic and Statistical Manual of Mental Disorders version 5), often accompanied by a high prevalence (83%) of co-occurring health and mental health conditions,^24^ which can interfere with building their own social networks. Social isolation is a common experience for autistic young adults^25^ and can adversely impact mental health,^26–29^ making it a critical target for intervention. Based on network approaches, we know that children on the autism spectrum have fewer connections with their classmates, fewer friendship nominations, lower levels of reciprocity with peers,^30–32^ and are more likely to be on the periphery in social groups.^30,31,33–36^ Seeking out friendship and social connections with peers in young adulthood can require resilience and persistence in the face of past difficulties. Stigmatization^37^ can also further isolate autistic young adults, as social capital theory suggests that people evaluate potential exchange partners based on their ability to reciprocate in future, referred to as a “credit slip.”^5^ Autistic young adults might be perceived by others as having less ability to reciprocate in future social exchanges. Social network approaches may illuminate these relationship dynamics and social capital for autistic young adults. Although some research suggests that caregivers, teachers, therapists, and peers in a youth's network can broker connections with others on behalf of the youth,^36,38,39^ no studies have used network analysis to systematically measure how key ecosystem members in the networks of young adults might connect them to interpersonal relationships and social capital that could potentially improve their adult outcomes over the life course.

### Organizational effects on the social capital

Social capital emerges when young adults access the knowledge, skills, and resources embedded in network connections. Work, school, and other community institutions provide organizational and institutional opportunities that facilitate network connections. Instead of using personal resources to create opportunities to meet people and build networks, people can gain connections by participating in organizational events, routines, and every day activities.^7^ Sociability in the workplace kitchen at lunch, repeated contact through work groups, “happy hour” after work with colleagues, and clubs and activities (e.g., fraternities, intermural sports, and affinity groups) all represent informal and formal opportunities to build interpersonal relationships as part of work or college life. Disconnection from these adult institutions could create less diverse networks for autistic people. Specifically, autistic young adults may have fewer connections with people outside the home/family: friends, romantic partners, coworkers, colleagues, coaches, and counselors affiliated with workplaces or educational institutions. Less diverse networks can limit social capital exchange opportunities, reducing exposure to different resources offered by diverse types of groups.^8^ Network data could characterize the strengths, gaps, and opportunities in autistic young adult social networks and how they vary across organizational contexts, providing targets for potential intervention that could protect against adverse outcomes.

### The “service cliff” and disparities in social capital

In addition to disconnection from work and school, autistic young adults can also experience a “service cliff”^1^ involving disruptions in access to needed supports as they age out of eligibility for school-based and pediatric care. Rates of service utilization decrease considerably after high school exit, and over one-quarter of autistic young adults received no services between high school and their early 20s (Ref.^1^) For those who have had some abiding continuity of care during adolescence in a given school or pediatric setting, transition out of these settings often also represents a loss of social capital in the form of trusting relationships, shared expectations, helpful resources, and useful knowledge that could connect them with opportunities for employment, continued learning, and community participation that fit their unique profile of strengths and needs.^3–7^ Autistic young adults living independently in the community report smaller networks, restricted access to instrumental and informational support, and greater dissatisfaction with their networks when compared with typically developing peers.^40^

### Gender differences in social capital

Social interactions of those on the autism spectrum vary by gender. Autistic women and girls tend to show higher social motivation and friendship formation.^41,42^ This could shape gender differences in social capital, with women developing more social connections than men. However, autistic women often receive a diagnosis and accompanying supports later than men.^43^ Later diagnosis could adversely impact connections with service providers, with earlier diagnosis providing advantages for developing more robust professional supports. Alternatively, later diagnosis or less interaction with professional supports may increase social engagement, as youth with less professional support might rely more on others in their community for natural supports and nonprofessional interpersonal relationships. In addition, research on nonautistic adults suggest that women form networks with more family kin relations than men,^44^ but this has not been tested among autistic young adults. Furthermore, there is very limited data on the social capital or networks of transgender or gender nonconforming individuals, who represent up to 22.1% of autistic survey respondents in some studies.^45^ Egocentric network data can reveal the composition of family versus nonfamily alters that make up the social networks of autistic young adults and investigate whether social closure between family and nonfamily alters varies by gender.

### Family members as key brokers

Although connections to peers, friends, romantic partners, coworkers, job coaches, job supervisors, service providers, fellow students, or college faculty can provide access to helpful resources, family members are also a key source of social support and job supports for people with disabilities.^16,46^ Strong family ties for adults with disabilities are positively associated with job satisfaction^46^ and the number of family-of-origin ties is associated with employment quality indicators (e.g., hours worked).^16^ The ecosystem of family support, broader community resources, and workplace capacity building supports are all interconnected to job readiness and employment for autistic young adults.^47^ However, over-reliance on family connections might also restrict the development of nonfamily connections. During childhood, parents can build networks with school staff members and community providers to increase their access to potential social capital for their autistic child.^48^ No research studies have investigated how the networks of autistic young adults and their parents interrelate. Investigating network connections across the ecosystem will provide important insight into how interpersonal relationships at home and outside of the home shape adult outcomes for autistic young adults.

Despite compelling possibilities for the application of social network analysis to the field of autism, progress investigating interactions and the influence of various types of social capital in the ecosystems of autistic young adult has been limited in several ways. Autism research has primarily relied on clinical interventions that teach social skills, rather than investigating how contexts shape the formation of relationships for autistic young adults. We have much to learn about how interpersonal relationships emerge for young adults on the spectrum. Few studies investigate how ecosystem members, such as parents and providers, establish their own connections to support the transition successfully to adulthood, or how they might facilitate connections between autistic young adults and others. The investigation of how autistic young adults themselves find, access, and utilize social capital available outside of their family is underinvestigated. Currently, there is limited data on racial or socioeconomic disparities in autistic adult outcomes, and network studies can investigate how inequalities in access to social capital may contribute.^49^ One feasibility study, conducted with college-going autistic young adults, provided preliminary support for the use of network measures for perceived support during college.^50^

In this article, we discuss the importance of relationships and social capital for autistic young adults and propose social network approaches to mathematically measure them. To demonstrate how to do this, we describe a social network protocol designed to map the networks of autistic young adults and systematically characterize their interpersonal relationships and social capital. We demonstrate how sociograms can be used to illustrate egocentric and duocentric networks. Finally, we recommend protocol adaptations and revisions to more accurately capture the ecosystem of connections of autistic young adults for future studies.

## Methods

### Subject protection

This study was approved by the official institutional review boards at Drexel University where these data were collected, analyzed, and published.

### Social network approaches for autism and adulthood

The primary aims of this article are to (1) demonstrate the potential contribution of social network measures to the field of autism to better capture social capital inequalities in adulthood and (2) provide an illustration of how to apply such methods. The egocentric and duocentric network methods and the data analyzed for this article are for illustrative and feasibility purposes only. A larger study with a statistically viable sample is required to determine how social networks are associated with outcomes.

### Study participants

The network feasibility data analyzed for this article were part of a qualitative study of autistic young adults (*n* = 20) and parents of autistic young adults (*n* = 21) about outcomes after high school. From the larger study, 17 young adults (ages 18–29 years) and 3 parents from matched youth–parent pairs were asked to complete a questionnaire covering demographics, service access, and employment, and egocentric social network. Young adults were predominantly white (67%), with a mean age 23.25 years (standard deviation = 3.6). Of them, 59% were men (*n* = 10, mean age = 21.78 years), 29% were women (*n* = 5, mean age = 25.2 years), and 12% were gender nonconforming (*n* = 2, mean age = 25 years). Young adults were situated in relatively advantaged families, with high levels of maternal 4-year college attainment (71%, *n* = 12), including 35% with a graduate degree or higher (*n* = 6). Overall, 82% of young adults had ever attended college and 35% were living independently (*n* = 6) (Table 1).

**Table 1.** Young Adult Study Participant Characteristics (*N* = 17)

	Mean
Age	23.25
White, %	67
Attended any college, %	82
Maternal graduate degree, %	35
Living independently, %	35
Dating, %	18
Employed, %	29

The autistic young adults who participated in this study lived in a major metropolitan area in the northeastern United States and were recruited using community contacts and snowball sampling. Young adults provided self-report of their Autism Spectrum Disorder (ASD) diagnosis. All young adult participants were their own legal guardian. Each participant received a $50 gift card.

### Egocentric social network measure

Social network measurement includes the number and type of social connections a person has with others.^20,51,52^ Egocentric networks include the respondent (“ego”) and key people whom the respondent identifies (“alters”).^53^ A “name generator” question^54^ was asked, and for each name generated, several additional questions were asked that characterize the alters in each participant's network. For this study, participants were asked to identify important people in their lives with the following name generator: “Please name up to five people who are very important to you.” The most important person was identified first, then the second most important until the youth stopped naming people or five people were named. This relationship represents a strong tie between the ego and the identified alters. For each of the people named, additional questions focused on their role in the participant's life (mother, father, friend, neighbor, therapist, etc.) and the supports that each alter provided. Support was calculated by dividing the total number of alters who provided a particular type of support by the total number of alters named. The young adult also identified connections among alters. For each unique pair of alters, the young adult was asked the network generator, “Does (Person A) interact with (Person B)? (If yes) How often?” The name generator was limited to five people to reduce burden on the participant, as each additional person named generates additional attribute and connection questions. The egocentric network survey developed for this study had 41 questions.

To produce duocentric networks that combine parent and young adult networks, parents were asked the following name generator: “Please name up to five people who currently help you support your young adult on the autism spectrum.” Parents were also asked to identify the role of each alter, the types of support alters provided for the parent to help their young adult, and whether alters had interacted with one another during the past year. This method has previously been applied to investigate the social networks of married couples^22^ and professor–student pairs^23^ and some adaptations were necessary.

### Duocentric networks

For three matched pairs, alter networks for both the young adult and the parent were calculated, and duocentric networks were created. If parents were named by the young adult as one of their alters, they were included as a node in the participant's network. In data from the matched pairs, all identified alters were sorted on a spreadsheet and manually coded for potential parent–young adult matches by first and last name. Other characteristics (i.e., role) were used as needed to finalize match coding. Once matching alters had been identified, unique identifiers were created for each alter. Following Kennedy et al.,^22^ reported ties among same alters were compared. Mismatches, such as one member of the pair reporting that alters had interacted while the other member reported that they had not, were noted. Among the three matched pairs, only one tie was discrepant. In the sociogram, we represented the network with the maximum value, which in this case was the reported tie by the parent.^22^

Six overall network measures were calculated for duocentric networks, including network size (the number of alters included in the network), network density (the number of actual ties between alters divided by the number of possible ties), network overlap members (the number of alters named by both parent and young adult), network unique members (the number of alters named by only the parent or young adult), disconnected network members (the number of alters named who were not tied to other alters), and parent centrality (the number of alters tied to the parent divided by the total number of alters minus the parent). Five network characteristics were calculated, including whether young adults named alters that the parent did not name, whether parents named alters that the young adult did not name, whether the parent network was a subset of the young adult network, whether overlap alters were tied to most other alters (coded as “yes” if 75% or more of alters were tied to the alter that was named by both the youth and the parent), and whether parents were tied to most other alters (coded as “yes” if 75% or more of alters were tied to the parent). Social capital measures were calculated to determine the social capital configuration for each matched pair.

### Network visualizations

**FIG. 1.**
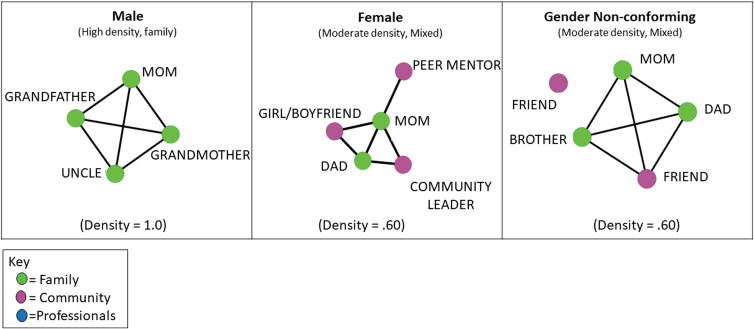
Representative alter egocentric networks by gender type.

**FIG. 2.**
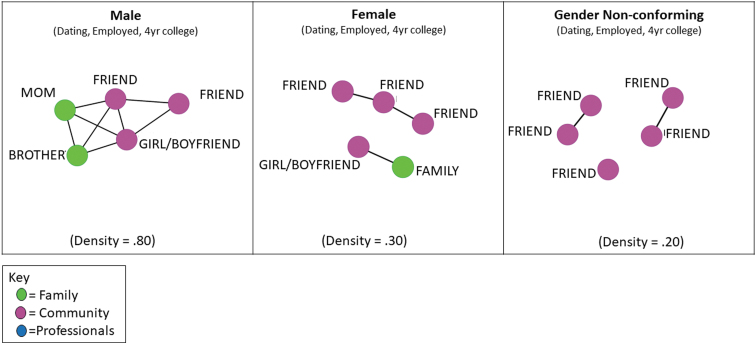
Alter egocentric networks of dating young adults.

**FIG. 3.**
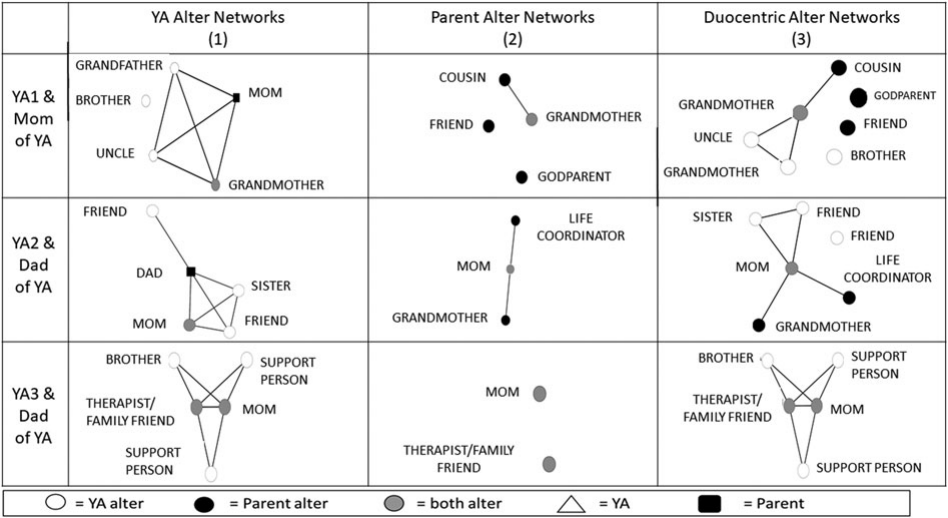
Matched pair networks of young adults and parents. YA, young adult.

Visualizations assist in the analysis of social networks.^55,56^ Visualizations of networks are provided to demonstrate what a network looks like and how each young adult's data are represented in a sociogram. All networks illustrated in this article were configured using a multidimensional scaling algorithm that positions nodes closer together that share similar connections. To illustrate various network configurations, we visualize selected example networks that represent different structural and/or compositional characteristics. Figures 1 and 2 illustrate representative young adult networks. All alter networks for the three matched pairs are illustrated in Figure 3. We colored the nodes to illustrate the different roles of alters (i.e., family, community, and professional).

### Feasibility data analysis

Data were collected through Qualtrics, and R was used to reconfigure the Qualtrics file into a social network edge list to compute social network measures. R was used to create an analytical database with all variables produced by the network survey. The data were visualized using ORA social network analysis software.^57^ These data are provided to illustrate the types of variables produced by the measure, the visualizations of various network types, and to present lessons learned from this feasibility study to support further research using social networks methods.

## Lessons Learned from the Social Network Feasibility Study

Although several findings from the study suggested that some aspects of the egocentric survey as implemented were feasible, other findings suggest revisions to the protocol and analyses that will yield more accurate and robust measurement. Figures 1–3 include gender identification to demonstrate the feasibility of comparing network variation across gender categories rather than to present generalizable results. We detail lessons learned from the feasibility study hereunder.

### Participant burden

Our network survey was purposefully designed to minimize participant burden by limiting the roster to the young adult's five most important alters. We modeled our survey after national surveys that used egocentric network generators of five alters [i.e., general social survey (GSS), national social life, health, and aging project]. In the GSS, a national sample of adults reported an average of 3.01 people with whom they discussed important matters in the past 6 months, suggesting that the five-person cap was not a constraint for the average participant.^58^

Despite the reduced number of alters, the respondent burden for naming five alters is still high, with a total of 41 responses required per participant to capture the network data presented in this article. Although other studies that have allowed for 20 or more named alters have reported means between 12 (Ref.^59^) and 18.5 (Ref.^60^), we selected a more restricted roster partly due to participants having completed an 85-question demographics, services, and employment questionnaire before completing network measures. Implementation analysis showed that all young adult and parent participants who attempted the social network survey completed all 41 questions, demonstrating good feasibility.

### Characterization of network size and roles

Compositional and structural variables were successfully calculated from the egocentric survey (Table 2). While a statically viable sample is required to provide accurate estimates of network size, density, and composition, Table 2 shows the variables produced by the social network measure to characterize young adult networks. The average network size for autistic young adults in this feasibility study was 4.88 and the median value was 5, suggesting that the young adults in our study were likely constrained by the five-person cap. This indicated that our strategy for reducing burden might result in the inaccurate characterization of the social networks of autistic young adults. Lifting the five-person cap and adding additional question probes by role type would likely provide a more accurate characterization of the social ecosystem of the young adult.

**Table 2.** Social Network Measures of Young Adult Networks (*N* = 17)

	Mean
YA alter networks	
YA network size	4.88^a^
Network density	0.67^b^
Team roles^c^	
% Family members	61
% Community members	36
% Professional providers	4

^a^ Average number of important people named by the young adult.

^b^ Density = 3 of actual ties/number of total possible ties.

^c^ Average percentage of each role type named as important people by the young adult.

YA, young adult.

The majority of important people named were family (61%), followed by community members (36%), with very few service providers named as alters. If the five-person cap was lifted, important people outside of the home/family might have been named. Future protocols with increased or minimal limits for young adults would likely capture a more accurate description of social capital resources. However, respondent burden and cost are often a concern with social network protocols, making open-ended generators challenging to implement.

### Characterization of network social capital

The types of social capital embedded in the networks were also successfully derived from the egocentric survey (Table 3). For the small sample of young adults who participated in the feasibility study, the most commonly accessed supports were friendship (75%), emotional support (74%), and advice (73%), whereas the least commonly accessed was employment support (24%). Although this sample showed robust family support, family alters were infrequent providers of employment support.

**Table 3.** Types of Social Capital Embedded in Young Adult Networks (*N* = 17)

	Mean
% Friendship	75
% Family friendship^a^	43
% Emotional support	75
% Advice	73
% Financial support	34
% Logistical support	31
% Job support	24

^a^ Average percentage of family members who provided the young adult with friendship.

Family friendship also varied by gender, where men had on average a marginally greater percentage of family members who provided friendship (mean = 58%) than women (mean = 32%) and gender nonconforming young adults, who reported no family friendship (mean = 0%). This suggests that gender is a salient variable in research on the acquisition of social capital through diversified networks, and that inclusive measures of gender identity are an important component of future research.

### Characterization of network variation by key attributes

Social network analysis is often used to measure variation by key demographic attributes, such as race, ethnicity, age, and gender. In the field of autism, variation across gender is likely related to networks. Although much research in sociology and related fields suggests that social networks vary by gender,^61,62^ our pilot sample is too small to allow for an accurate test of differences across gender. For example, we cannot control for key variables that might also impact network formation, such as age. The mean age for males is 21 years in our sample, whereas mean age for females is 25 years, likely impacting social network formation in important ways. Although a statically viable sample with enough power to control for key demographic variables is needed to confirm findings, Figure 1 is used in this article to demonstrate how to interpret variation across social network configuration and produce comparisons across key attributes. Upon examination of the sociograms of egocentric networks, the egocentric networks of both men and women were characterized by complete social closure among family members. Women's networks were characterized by the inclusion of more community members, who had fewer connections with each other, on average, and fewer connections with the woman's family members (see Fig. 1 for representative networks). In the male network, all important people, including mother, uncle, grandfather, and grandmother, are tied to each other. In contrast, in the female network, family members are tied to each other and to community members, with no connections between community members. The gender nonconforming sociogram shown in Figure 1 has complete social closure for family members and one community member, with mother, father, brother, and friend all tied to each other and no connections between the second friend and any other member of the network. The presence of community members as important people in the networks of females and gender nonconforming young adults provides a qualitative indication of potentially reciprocal relationships established outside of the family. In future studies, social isolation can be contextualized through the use of social network measures like the one proposed in this article. Additional questions that ask young adults to rate the degree of reciprocity with alters would also provide a useful measure of relationship quality.

Emerging research suggests that autism diagnosis is associated with higher rates of being transgender^63^ or of rejecting a binary gender identity.^64,65^ Gender nonconforming young adults were present in the sample in small numbers (*n* = 2). The two particular gender nonconforming young adults in our study had networks that were more similar to women's networks, with one young adult identifying only friends and no family as alters. This was the only network that did not include any family, and future research on autism and networks could investigate whether there are network differences for transgender/gender nonconforming young adults and whether such differences are associated with social stigma at home.^37^

Recent research suggests that the majority of adolescents and adults on the autism spectrum without intellectual disability engage in sexual relationships, in contrast to past research identifying sexuality as a problematic issue for people on the autism spectrum.^66–68^ Few participants in this sample were dating (*n* = 3), and all participants who were dating were also employed, attended a 4-year college, and had networks primarily made up of friends (Fig. 2). The sociograms shown in Figure 2 represent the egocentric networks of the three young adults in the sample who were dating.

### Feasibility of parent/young adult duocentric network method

Paired network measures offer new ways to investigate the role that parents play for young people during and after the transition to adulthood. Table 4 illustrates combined duocentric network data for parent–young adult dyads. Alter network size and density varied by young adult–parent matched pair. Two young adults experienced increased ecosystem network size and one experienced no change in ecosystem network size (YA3). Network density between the young adult and the parent for both YA1 and YA3 varied significantly for different reasons. YA1's parent identified three unconnected alters who helped them support their young adult, resulting in a network wherein only 17% of all possible alter connections were tied. In comparison, YA1 identified only one alter who was not tied to others, resulting in a network wherein 60% of all possible alter connections were tied (see Fig. 3 for illustration). YA3's parent identified two alters who were not tied, yielding a density score of 0, whereas 70% of possible connections among YA3's alters were tied. It is important to note that YA3 and her parent disagree about the connection between the two alters named by the parent. The parent reports the pair as unconnected and the young adult reports the same pair as connected. For duocentric network analysis, maximum value is used when combining all alters together.

**Table 4.** Matched Young Adult and Parent Alter Social Networks

	YA1-PA (Male)		YA2-PA (Male)		YA3-PA (Female)	
	YA alter	PA alter	YA alter	PA alter	YA alter	PA alter
	(1)	(2)	(1)	(2)	(1)	(2)
Overall network measures						
Network size	5	4	5	3	5	2
Network density	0.6	0.17	0.7	0.67	0.7	0
Network overlap	1	1	1	1	2	2
Network unique	3	3	3	2	3	0
Network disconnected	1	2	0	0	0	2
Parent centrality	75%		100%		NA	
Network characteristics						
Parent only alters	Yes		Yes		No	
Parent high centrality	Yes		Yes		No	

NA, parent not included in YA network; PA, parent.

All matched pair alter networks had at least one member that overlapped with both alter networks. All but one parent alter network (YA3's parent) identified unique alters. Only one young adult network had disconnected alters (YA1) and two parent networks had disconnected alters (PA1 and PA3). Parent centrality was high (>75% of alters were connected to the parent) from two networks (YA1 and YA2); however, YA3 did not name their parent as one of their top five important people, so parent centrality was not calculable. Two types of matched networks emerged from the analysis, including combined networks wherein parents occupied a central position in young adult alters and added new alters (YA1 and YA2) and an alter network wherein the parent's alters were a subset of the young adult's alters (YA3). The study findings suggest that duocentric network analysis is a feasible method for investigating the degree of brokerage that parents provide autistic young adults to social capital outside the immediate sphere of the young adult networks.

Although we were successfully able to code the network data and prepare duocentric networks for each matched pair as proposed by Kennedy et al.^22^ (Fig. 3), we discovered a few key issues that interfered with our efforts to generate accurate duocentric networks.

First, we imposed a five-person limit on the roster generator. This is problematic for duocentric networks because it is possible that the young adult and the parent will name a different set of people for their top five, whereas a longer list would elicit more overlap. In addition, using the same name generator rather than “important people” (young adults) and “important people who help you support young adult” (parent) may result in different overlap in duocentric networks.

### Characterization of social capital in parent/young adult duocentric network

Parent alter networks most often added more familial and community members with additional friendship, emotional support, and advice (YA1 and YA2). YA2's joint resources did include added logistical and job support from the parent's alters. YA3 did not receive any additional resources from the parent alter network, since it was a subset of the young adult's network (Table 5).

**Table 5.** Matched Young Adult and Parent Alter Social Capital

	YA1-PA (Male)			YA2-PA (Male)			YA3-PA (Female)		
	YA alter, %	PA alter, %	#	YA alter, %	PA alter, %	#	YA alter, %	PA alter, %	#
	(1)	(2)	ADD	(1)	(2)	ADD	(1)	(2)	ADD
Network roles									
% Family	100	75	1	40	50	1	60	67	0
% Community	0	25	2	0	0	0	40	0	0
% Professional	0	0	0	60	50	1	0	33	0
Network support and resources									
% Friendship	100	50	1	100	100	2	40	67	0
% Family friendship	100	25	0	40	50	1	0	33	0
% Emotional support	60	100	3	80	100	2	20	67	0
% Advice	40	50	2	80	100	2	0	67	0
% Financial support	40	0	0	60	50	0	20	33	0
% Logistical support	20	0	0	60	50	1	60	67	0
% Job support	20	0	0	60	50	1	20	67	0

ADD, additional number of roles/support and resources provided by parent alters unique to parent network.

### Network study design

Several other aspects of our study protocol require revision to better characterize social capital for autistic young adults. First, including young autistic adults as advisors on the development of a social network survey through community-based participator research^69,70^ would yield valuable insights for measure development.

Importantly, the study did not include baseline measurement of networks before youth transitioned from high school, making it difficult to discern mechanisms in young adult networks that drive changes and differences post-transition. Further specification of types of employment supports is needed, including discernment of potential organizational sources of social capital related to employment, such as participation in college programs that facilitate employment networks through internships or enrollment in state and federal funding programs for vocational rehabilitation supports for employment coaching and training. Social network research investigating how pre-employment interventions impact network formation related to employment is needed.

The current sample also lacks variation across socioeconomic status, racial, ethnic diversity, and functionality, suggesting the need for further investigation regarding feasibility for less resourced more diverse populations. Comparison research investigating variance in employment social capital embedded in autistic adult networks as compared with those of nonautistic adults would yield valuable information about outcomes disparities. In addition, determining which network configurations are associated with improved young adult outcomes will require appropriately powered samples.

## Conclusions

This study confirms the feasibility for utilizing social network approaches with autistic young adults to capture social connectivity, including density and distribution of network roles, and to track access to social capital resources through networks. This provides information about mutable factors. Future research with an appropriately powered sample can determine whether variation in social networks and access to social capital are associated with better outcomes. Identifying mutable network factors will guide interventions that foster network configurations to improve outcomes for young adults on the spectrum.^71^

## Authorship Confirmation Statement

E.M.H. conceptualized and designed the social network components of the study, analyzed and interpreted the network data, and drafted the initial article with findings. C.S. and P.T.S. conceived of the overarching qualitative study. C.S. collected data and oversaw research assistants who participated in data collection. L.A.H. assisted in drafting the article. J.W. conducted literature reviews and supported analysis and preparation of references. E.M.H. drafted this article with input on revisions from C.S., L.G.H., and P.T.S. All coauthors have reviewed and approved of the article before submission. The article has been submitted solely to this journal and is not published, in press, or submitted elsewhere.

## References

[1] Roux AM, Shattuck PT, Rast JE, Rava JA, Anderson KA National autism indicators report: Transition into young adulthood. *Life Course Outcomes Research Program*, AJ Drexel Autism Institute, Drexel University, Philadelphia, PA, 2015

[2] Shattuck PT, Narendorf SC, Cooper B, Sterzing PR, Wagner M, Taylor JL Postsecondary education and employment among youth with an autism spectrum disorder. Pediatrics. 2012;129(6):1042–1049 22585766 10.1542/peds.2011-2864 PMC3362908

[3] Bourdieu P Cultural reproduction and social reproduction. In: Grusky DB, Szelenyi S, eds. Knowledge, Education, and Cultural Change. New York: Routledge; 1977:71–112

[4] Coleman JS. Social capital in the creation of human capital. Am J Soc. 1988;94:S95–S120

[5] Coleman JS. Foundations of Social Theory. Cambridge, MA: Belknap; 1990

[6] Lin N. *Social Capital: A Theory of Social Structure and Action.* Vol. 19. Cambridge, UK: Cambridge University Press; 2001

[7] Small ML. Unanticipated Gains: Origins of Network Inequality in Everyday Life. Oxford, UK: Oxford University Press; 2009

[8] Adler PS, Kwon S-W Social capital: Prospects for a new concept. Acad Manag Rev. 2002;27(1):17–40

[9] Chen M-J, Su K-H, Tsai W Competitive tension: The awareness-motivation-capability perspective. Acad Manag J. 2007;50(1):101–118

[10] Maurer I, Bartsch V, Ebers M The value of intra-organizational social capital: How it fosters knowledge transfer, innovation performance, and growth. Organ Stud. 2011;32(2):157–185

[11] Moody J, Paxton P Building Bridges: Linking Social Capital and Social Networks to Improve Theory and Research. Los Angeles, CA: Sage Publications Sage CA; 2009

[12] Burt RS The social structure of competition. In: Cross R, Parker A, Sasson L, eds. Networks in the Knowledge Economy. Oxford: University Press; 1992;13–56

[13] Granovetter MS. The strength of weak ties. In: Social Networks. Elsevier; 1973:347–367

[14] Lin N, Dumin M Access to occupations through social ties. Soc Netw. 1986;8(4):365–385

[15] Lin N, Ensel WM, Vaughn JC Social resources and strength of ties: Structural factors in occupational status attainment. Am Soc Rev. 1981;46:393–405

[16] Baldridge D, Konrad AM, Moore ME, Yang Y Childhood-onset disability, strong ties and employment quality. Equal Divers Inclus Int J. 2017;36(4):290–305

[17] Burt RS, Talmud I Market niche. Social Networks 1993;15:133–149

[18] Hedberg E. Dyad vs. network effects: Modeling relationships in personal networks using contextual effects. *Soc Sci Res.* 2017;63:339–355 28202153 10.1016/j.ssresearch.2016.08.020

[19] Mortensen DT, Pissarides CA Job creation and job destruction in the theory of unemployment. Rev Econ Stud. 1994;61(3):397–415

[20] Wasserman S, Faust K *Social Network Analysis: Methods and Applications.* Vol. 8. Cambridge, UK: Cambridge University Press; 1994

[21] Lord C, McGee J Educating Children with Autism. Washington, DC: National Academy Press; 2001

[22] Kennedy DP, Jackson GL, Green HD, Bradbury TN, Karney BR The analysis of duocentric social networks: A primer. J Marriage Fam. 2015;77(1):295–311 27182084 10.1111/jomf.12151 PMC4864858

[23] Coromina L, Guia J, Coenders G, Ferligoj A Duocentered networks. Soc Netw. 2008;30(1):49–59

[24] Levy SE, Giarelli E, Lee L-C, et al. Autism spectrum disorder and co-occurring developmental, psychiatric, and medical conditions among children in multiple populations of the United States. J Dev Behav Pediatr. 2010;31(4):267–275 20431403 10.1097/DBP.0b013e3181d5d03b

[25] Lounds Taylor J, Adams RE, Bishop SL Social participation and its relation to internalizing symptoms among youth with autism spectrum disorder as they transition from high school. Autism Res. 2017;10(4):663–672 27739234 10.1002/aur.1709 PMC5392176

[26] Bagwell CL, Newcomb AF, Bukowski WM Preadolescent friendship and peer rejection as predictors of adult adjustment. Child Dev. 1998;69(1):140–153 9499563

[27] Bauminger N, Kasari C Loneliness and friendship in high-functioning children with autism. Child Dev. 2000;71(2):447–456 10834476 10.1111/1467-8624.00156

[28] Bukowski WM, Laursen B, Hoza B The snowball effect: Friendship moderates escalations in depressed affect among avoidant and excluded children. Dev Psychopathol. 2010;22(4):749–757 20883579 10.1017/S095457941000043X

[29] Matthews T, Danese A, Wertz J, et al. Social isolation, loneliness and depression in young adulthood: A behavioural genetic analysis. Soc Psychiatry Psychiatr Epidemiol. 2016;51(3):339–348 26843197 10.1007/s00127-016-1178-7 PMC4819590

[30] Locke J, Ishijima EH, Kasari C, London N Loneliness, friendship quality and the social networks of adolescents with high-functioning autism in an inclusive school setting. J Res Spec Educ Needs. 2010;10(2):74–81

[31] Kasari C, Locke J, Gulsrud A, Rotheram-Fuller E Social networks and friendships at school: Comparing children with and without ASD. J Autism Dev Dis. 2011;41(5):533–544 10.1007/s10803-010-1076-x PMC3076578 20676748

[32] Locke J, Anderson A, Frederick L, Kasari C Understanding friendship sex heterophily and relational characteristics to optimize the selection of peer models for children with autism spectrum disorder. J Autism Dev Dis. 2018;48(12):4010–4018 10.1007/s10803-018-3662-2 PMC6219927 29982894

[33] Locke J, Kasari C, Rotheram-Fuller E, Kretzmann M, Jacobs J Social network changes over the school year among elementary school-aged children with and without an autism spectrum disorder. School Mental Health. 2013;5(1):38–47

[34] Chamberlain B, Kasari C, Rotheram-Fuller E Involvement or isolation? The social networks of children with autism in regular classrooms. J Autism Dev Dis. 2007;37(2):230–242 10.1007/s10803-006-0164-4 16855874

[35] Rotheram-Fuller E, Kasari C, Chamberlain B, Locke J Social involvement of children with autism spectrum disorders in elementary school classrooms. J Child Psychol Psychiatry. 2010;51(11):1227–1234 20673234 10.1111/j.1469-7610.2010.02289.x PMC2970745

[36] McGhee Hassrick E, Shattuck P, Carley K Network measures of collaborative support for young adults with autism. Pediatrics. 2018;141(Suppl. 4):S287–S292 29610409 10.1542/peds.2016-4300E PMC5877126

[37] Gray DE. “Everybody just freezes. Everybody is just embarrassed”: Felt and enacted stigma among parents of children with high functioning autism. *Sociol Health Illness.* 2002;24(6):734–749

[38] Kreider CM, Bendixen RM, Young ME, Prudencio SM, McCarty C, Mann WC Social networks and participation with others for youth with learning, attention, and autism spectrum disorders: Réseaux sociaux et participation avec les autres, chez des adolescents ayant des troubles d'apprentissage, de l'attention et du spectre de l'autisme. Canad J Occupat Ther. 2016;83(1):14–26 10.1177/0008417415583107 PMC4710853 26755040

[39] Calder L, Hill V, Pellicano E “Sometimes I want to play by myself”: Understanding what friendship means to children with autism in mainstream primary schools. Autism. 2013;17(3):296–316 23188883 10.1177/1362361312467866

[40] Asselt-Goverts AEv, Embregts PJCM, Heniks AHC, Wegman KM, Teunisse JPWM Do social networks differ? Comparison of the social networks of people with intellectual disabilities, people with autism spectrum disorders and other people living in the community. J Autism Dev Dis. 2015;45(5):1191–1203 10.1007/s10803-014-2279-3 PMC4544488 25326258

[41] Head AM, McGillivray JA, Stokes MA Gender differences in emotionality and sociability in children with autism spectrum disorders. Mol Autism. 2014;5(1):19 24576331 10.1186/2040-2392-5-19 PMC3945617

[42] Sedgewick F, Hill V, Yates R, Pickering L, Pellicano E Gender differences in the social motivation and friendship experiences of autistic and non-autistic adolescents. J Autism Dev Disord. 2016;46(4):1297–1306 26695137 10.1007/s10803-015-2669-1 PMC4786616

[43] Giarelli E, Wiggins LD, Rice CE, et al. Sex differences in the evaluation and diagnosis of autism spectrum disorders among children. Disabil Health J. 2010;3(2):107–116 21122776 10.1016/j.dhjo.2009.07.001 PMC4767258

[44] Moore G. Structural determinants of men's and women's personal networks. Am Sociol Rev. 1990;55(5):726–735

[45] Dewinter J, De Graaf H, Begeer S Sexual orientation, gender identity, and romantic relationships in adolescents and adults with autism spectrum disorder. J Autism Dev Dis. 2017;47(9):2927–2934 10.1007/s10803-017-3199-9 PMC5570786 28597143

[46] Pérez V, Alcover C-M, Chambel MJ Job attitudes among workers with disabilities: The importance of family support in addition to organizational support. Work. 2015;51(4):817–826 24962307 10.3233/WOR-141905

[47] Nicholas DB, Mitchell W, Dudley C, Clarke M, Zulla R An ecosystem approach to employment and autism spectrum disorder. J Autism Dev Dis. 2018;48(1):264–275 10.1007/s10803-017-3351-6 29071564

[48] McGhee Hassrick E. Mapping the social infrastructures of special education interventions across home and school settings using social network analytics. In: Turner-Vorbeck SST, ed. Handbook of Family, School, Community Partnerships in Education. Washington, DC: Johns Hopkins University Press; 2019

[49] Bishop-Fitzpatrick L, Kind AJ A scoping review of health disparities in autism spectrum disorder. J Autism Dev Dis. 2017;47(11):3380–3391 10.1007/s10803-017-3251-9 PMC5693721 28756549

[50] Lei J, Ashwin C, Brosnan M, Russell A Developing an online tool to measure social network structure and perceived social support amongst autistic students in higher education: A feasibility study. J Autism Dev Dis. 2019;49:3526–3542 10.1007/s10803-019-04070-5 PMC6667418 31119511

[51] Berkman LF, Glass T, Brissette I, Seeman TE From social integration to health: Durkheim in the new millennium. Soc Sci Med. 2000;51(6):843–857 10972429 10.1016/s0277-9536(00)00065-4

[52] Smith KP, Christakis NA Social networks and health. Annu Rev Sociol. 2008;34:405–429

[53] Marsden PV. Network data and measurement. Ann Rev Sociol. 1990;16(1):435–463

[54] Burt RS. Network items and the general social survey. Soc Netw. 1984;6(4):293–339

[55] Freeman LC. Visualizing social networks. J Soc Struct. 2000;1(1):4

[56] Healy K, Moody J Data visualization in sociology. Ann Rev Soc. 2014;40:105–128 10.1146/annurev-soc-071312-145551 PMC4203684 25342872

[57] Carley KM, Reminga J. ORA: Organization risk analyzer. In: [Technical report]/Carnegie Mellon University. School of Computer Science. [Institute for Software Research International]. Carnegie Mellon University, School of Computer Science, [Institute for Software Research International]; 2004

[58] Marsden PV. Core discussion networks of Americans. Am Sociol Rev. 1987;52:122–131

[59] Hall A, Wellman B Social networks and social support. 1985

[60] Fischer CS. To dwell among friends: personal networks in town and city. Chicago, IL: University of Chicago Press; 1982: http://www.loc.gov/catdir/enhancements/fy0608/81011505-t.html; Materials specified: Table of contents only http://www.loc.gov/catdir/enhancements/fy0608/81011505-t.html

[61] Benenson JF. Gender differences in social networks. J Early Adolesc. 1990;10(4):472–495

[62] Dykstra PA, de Jong Gierveld J Gender and marital-history differences in emotional and social loneliness among Dutch older adults. Canad J Aging. 2004;23(2):141–155 15334814 10.1353/cja.2004.0018

[63] Strang JF, Kenworthy L, Dominska A, et al. Increased gender variance in autism spectrum disorders and attention deficit hyperactivity disorder. Arch Sex Behav. 2014;43(8):1525–1533 24619651 10.1007/s10508-014-0285-3

[64] Kristensen ZE, Broome MR Autistic traits in an internet sample of gender variant UK adults. Int J Transgend. 2015;16(4):234–245

[65] Cooper K, Smith LG, Russell AJ Gender Identity in autism: Sex differences in social affiliation with gender groups. J Autism Dev Dis. 2018;48(12):3995–4006 10.1007/s10803-018-3590-1 PMC6223803 29705922

[66] Rosqvist HB. Becoming an “autistic couple”: Narratives of sexuality and couplehood within the Swedish autistic self-advocacy movement. Sexual Disabil. 2014;32(3):351–363

[67] Dewinter J, Vermeiren R, Vanwesenbeeck I, van Nieuwenhuizen C Autism and normative sexual development: A narrative review. J Clin Nurs. 2013;22(23–24):3467–3483 24112137 10.1111/jocn.12397

[68] Kellaher DC. Sexual behavior and autism spectrum disorders: An update and discussion. Curr Psychiatry Rep. 2015;17(4):562 25749749 10.1007/s11920-015-0562-4

[69] Nicolaidis C, Raymaker D, McDonald K, et al. Collaboration strategies in nontraditional community-based participatory research partnerships: Lessons from an academic–community partnership with autistic self-advocates. Prog Commun Health Partnersh. 2011;5(2):143–150 10.1353/cpr.2011.0022 PMC3319698 21623016

[70] Powers LE, Garner T, Valnes B, et al. Building a successful adult life: Findings from youth-directed research. Exceptionality. 2007;15(1):45–56

[71] Valente TW. Network interventions. Science. 2012;337(6090):49–53 22767921 10.1126/science.1217330

